# Identification of 4-carboxyglutamate residue sites based on position based statistical feature and multiple classification

**DOI:** 10.1038/s41598-020-73107-y

**Published:** 2020-10-09

**Authors:** Asghar Ali Shah, Yaser Daanial Khan

**Affiliations:** 1 Department of Computer Sciences, Bahria University Lahore Campus, Lahore, 25000 Pakistan; 2 University of Management and Technology, Lahore, 25000 Pakistan

**Keywords:** Biotechnology, Computational biology and bioinformatics, Computer science

## Abstract

Glutamic acid is an alpha-amino acid used by all living beings in protein biosynthesis. One of the important glutamic acid modifications is post-translationally modified 4-carboxyglutamate. It has a significant role in blood coagulation. 4-carboxyglumates are required for the binding of calcium ions. On the contrary, this modification can also cause different diseases such as bone resorption, osteoporosis, papilloma, and plaque atherosclerosis. Considering its importance, it is necessary to predict the occurrence of glutamic acid carboxylation in amino acid stretches. As there is no computational based prediction model available to identify 4-carboxyglutamate modification, this study is, therefore, designed to predict 4-carboxyglutamate sites with a less computational cost. A machine learning model is devised with a Multilayered Perceptron (MLP) classifier using Chou’s 5-step rule. It may help in learning statistical moments and based on this learning, the prediction is to be made accurately either it is 4-carboxyglutamate residue site or detected residue site having no 4-carboxyglutamate. Prediction accuracy of the proposed model is 94% using an independent set test, while obtained prediction accuracy is 99% by self-consistency tests.

## Introduction

Proteins are a key element of every cell necessary to build and repair tissues. They are macromolecules constructed using a chain of amino acid residues. Proteins exhibit numerous properties, they may work as hormones, enzymes or may be a part of structural cellular component. Among 20 common proteins, glutamic acid is an important protein with a wide range of functions. Specifically, it has role in proper functioning of central and the peripheral nervous system^1^.

**Figure 1:**
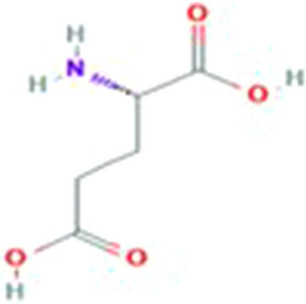
Structure of glutamic acid^9^.

**Figure 2:**
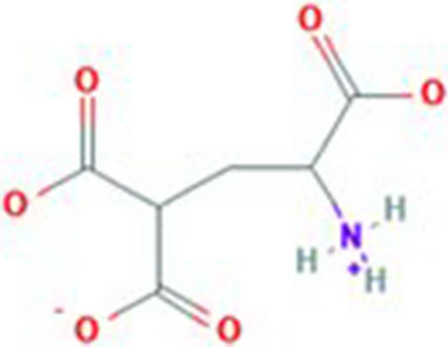
Structure of 4-carboxyglutamate^10^.

Vitamin K-dependent carboxylase is a bifunctional enzyme. It catalyzes the oxygenation of vitamin K hydroquinone, helps in formation of vitamin K epoxide, resulting the formation of carboxyglutamate. 4-carboxyglutamate is a modification of glutamic acid formed due to post-translational modification (PTM). The structure of glutamic acid and 4-carboxyglutamate is explained in Figs. 1 and 2. These modified residues are then further exploited to bind calcium ions. These calcium ions provide positive charges to glutamic acids which further interact with the negatively charged phospholipid membrane^2,3^. Carboxylation has role in blood clotting and other biological processes^4,5^. The deficiency of vitamin K also results in deficiency of protein S and C which also formulate a Moyamoya disease. Carboxylation of glutamic acid causes disorders including bone resorption, osteoporosis, papilloma and plaque atherosclerotic^6–8^.

Experimenting and identification of 4-carboxyglutamate residue sites at laboratory is costly and time-consuming. Therefore, it is necessary to formulate a computational model to identify 4-carboxyglutamate residue sites.

This study focuses on the post translational modification of glutamic acid into 4-carboxyglutamic acid within the glutamic acid domain modification. An accurate and efficient prediction model is devised to serve the purpose. The methodology is based on a Chou’s 5-step rule^11^. These rules serve as a benchmark for dataset collection, mathematical formulation of samples, prediction-algorithm, and cross-validation of results and the development of web server. This methodology is further carried out one by one in the above said sequential order.

## Materials and methods

**Figure 3:**
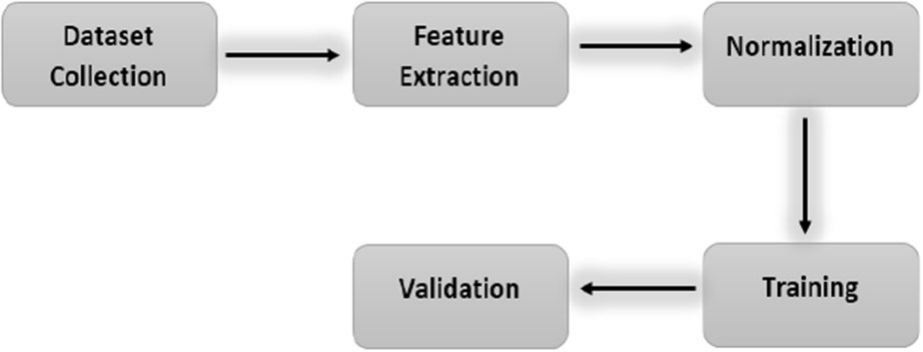
Flow chart of methodology.

Chou’s peptide formulation^11,12^ is widely used in many studies^13–19^. In this study, Chou’s formulation is also adopted to reach the solution. The operational flow chart of the chosen methodology is depicted in Fig. 3.

### Benchmark dataset

4-Carboxyglutamate sequences are extracted from a universal resource of protein (www.UniProt.org) through an advanced search query. The data is bifurcated as one with 4-carboxyglutamate modification and the other without 4-carboxyglutamate residues (also termed as positive and negative respectively). The redundancy and homology biases were excluded through CD-HIT web server (https://weizhongli-lab.org/cd-hit/) and the similarity threshold is 90%. Finally, a refined benchmark dataset of 261 proteins are constructed containing 560 positive and 600 negative samples. The total observations of obtained dataset are 560 + 600 = 1160. The dataset is represented by O. The positive observations are represented by O+, and negative observations within the data set are depicted by O−. U represents union according to the set theory.

1 $$ {\text{O }} = {\text{ O}}^{ + } {\text{U O}}^{ - } $$

### Sequence logo

**Figure 4:**
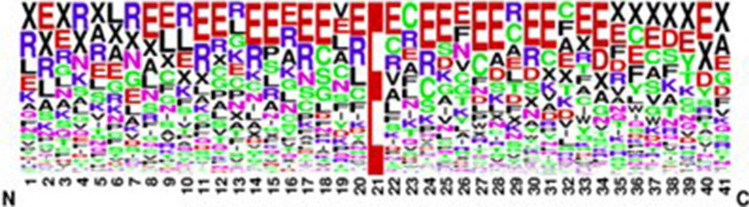
Sequence logo of positive 4-carboxyglutamate.

**Figure 5:**
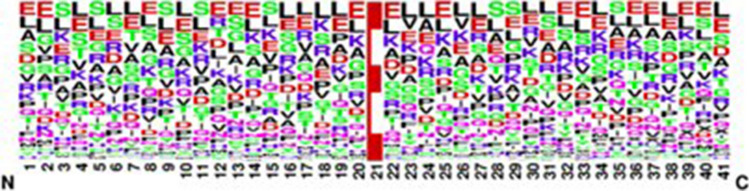
Sequence logo of negative 4-carboxyglutamate.

The PTM sequencing of the obtained dataset is graphically and visually represented in Figs. 4 and 5. Sequence conservation at a specific position is represented by the overall height of the stack.

### Sample formulation

The formulation of biological sequencing is one of the most critical problems in computational biology. Vector quantification is a key to formulate the sequence by maintaining their sequence patterns and features that are required for targeted analysis. As vector quantification paves a way for addressing the formulated sequencing using machine learning algorithms^20^. In this work, a pseudo amino acid composition (PseAAC)^21^ is chosen. According to the chosen composition, samples in the dataset can be described as^34^. Equation (2) depicts that each sample is a subsequence of fixed size while Eq. (3) depicts that 20 residues upstream and 20 residues downstream were extracted while R21 is the 4-carboxyglutamate site.

2 $$ {\varvec{B}}_{\xi = 7 } \left( {{\mathbb{K}}} \right) = \left[ {\Psi_{1} \Psi_{2} \ldots \Psi_{u} \ldots \Psi_{\Omega } } \right]^{T} $$

where u = 1, 2, 3 …Ω. It elaborates how useful features can be extracted from relevant peptide sequencing and T denotes transpose operator. Each sample peptide sequence is 41 in length due to which Eq. (2) can be formulated as.

3 $$ {\varvec{B}} = R_{1} R_{2} \ldots R_{19} R_{20} R_{21} \ldots R_{40} R_{41} $$

### Statistical moment calculation

The composition of each sequence of proteins follows some specific pattern. Due to such distinction, each sequence is to be described with different statistical parameters. In previous work, statistical moments are used for feature extraction^22,23^. In order to have feature extraction, raw, central and Hahn moments are used. The composition of amino acids has a very important role in the functionality and nature of the proteins. The extraction of the feature can be location and scale variant. To address location variant features, raw moments are used to calculate mean, variance and asymmetry of sample distribution in the dataset. Central moments are also used for feature extraction by estimating mean, variance and asymmetry but it is location invariant as the estimations are made using centroid but central moments are actually scaled variant^24,25^. Hahn moments are used to estimate statistical parameters but these moments are both location and scale variant^26,27^. Therefore Hahn moments are computed using Hahn polynomials to estimate the mean in dataset and variance in dataset and asymmetry of the probability distribution. For the said method, moments are computed in a two-dimensional n × n matrix denoted by B′^28^.

4 $$ {\mathbf{B}}^{\prime} = \left[ {\begin{array}{*{20}c} {b_{11} } & {b_{12} } & \cdots & {b_{1n} } \\ {b_{21} } & {b_{22} } & \ldots & {b_{2n} } \\ \vdots & \vdots & \ddots & \vdots \\ {b_{n1} } & {b_{n2} } & \cdots & {b_{nn} } \\ \end{array} } \right] $$

A function ω^29^ is a mapping function used for matrix transformation of **B** as **B**′. It uses the element from this matrix **B**′. Moments were computed up to order three such as M01, M10, M11, M12, M21, M30 and M03. The raw moments are computed as given below.

5 $$ M_{ij} = \mathop \sum \limits_{b = 1}^{n} \mathop \sum \limits_{q = 1}^{n} b^{i} q^{j} \beta_{bq} $$

The sum of i and j represents the order of the moments that is i + j and it can be less than or equal to three. The Central moments can be computed as given below.

6 $$ n_{ij} = \mathop \sum \limits_{b = 1}^{n} \mathop \sum \limits_{q = 1}^{n} \left( {b - \overline{x} } \right)^{i} \left( {q - \overline{y} } \right)^{j} \beta_{bq} $$

Hahn moments can be easily computed for even dimensional data organization. Reversible property of Hahn moments is evident due to their orthogonality^28^. Hahn moments of order n are computed as following,

7 $$ h_{n}^{u,v} \left( {r, N} \right) = \left( { N + V - 1 } \right)_{n} \left( {N - 1} \right)_{n} \times \mathop \sum \limits_{k = 0}^{n} \left( { - 1} \right)^{k} \frac{{\left( { - n} \right)_{k} \left( { - r} \right)_{k} \left( {2N + u + v - n - 1} \right)_{k} }}{{\left( {N + v - 1} \right)_{k} \left( { N - 1 } \right)_{k} }} \frac{1}{k!} . $$

Normalized orthogonal Hahn moments of two dimensional discrete are computed as

8 $$ H_{ij} = \mathop \sum \limits_{q = 0}^{N - 1} \mathop \sum \limits_{b = 0}^{N - 1} \beta_{ij} h_{i}^{{\widetilde{u,v}}} \left( {q,N} \right)h_{i}^{{\widetilde{u,v}}} \left( {b,N} \right) m, n = 0, 1, \ldots N - 1 .  $$

### Determination of PRIM and RPRIM

The primary sequence and relative position of residues are key factors to predict the characteristics of proteins. Quantitative characterization of the relative position of amino acid is also necessary. In order to serve the said purpose, 20 × 20 matrix is constructed as representative of Position relative Incidence Matrix (PRIM) to extract information about the relative position of each amino acid residue in the protein as given in Eq. (9).

9 $$ S_{PRIM} = \left[ {\begin{array}{*{20}c} {S _{1 \to 1} } & {S _{1 \to 2} } & {. \ldots } & {S _{1 \to j} } & {. \ldots } & {S _{1 \to 1} } \\ {S _{2 \to 1} } & {S _{2 \to 1} } & {. \ldots } & {S _{2 \to 1} } & {. \ldots } & {S _{2 \to 20} } \\ {S _{i \to 1} } & {S _{i \to 1} } & {. \ldots } & {S _{i \to 1} } & {. \ldots } & {S _{i \to 20} } \\ {S _{N \to 1} } & {S _{N \to 1} } & {. \ldots } & {S _{N \to 1} } & {. \ldots } & {S _{N \to 20} } \\ \end{array} } \right] $$

Information is extracted as 400 coefficients for PRIM. In order to reduce PRIM dimensionality, statistical moments are computed for PRIM which produces a set of 24 elements.

To make it more effective and better, identifying hidden features, Reverse Position Relative Incidence Matrix (RPRIM) is also computed as:

10 $$ S_{RPRIM} = \left[ {\begin{array}{*{20}c} {S_{ 1 \to 1} } & {S_{ 1 \to 2} } & \ldots & {S_{ 1 \to j} } & \cdots & {S_{ 1 \to 1} } \\ {S _{2 \to 1} } & {S_{ 2 \to 1} } & \ldots & {S_{ 2 \to 1} } & \ldots & {S_{ 2 \to 20} } \\ {S_{ i \to 1} } & {S_{ i \to 1} } & \ldots & {S_{ i \to 1} } & \ldots & {S_{ i \to 20} } \\ {S_{ N \to 1} } & {S_{ N \to 1} } & \ldots & {S_{ N \to 1} } & \cdots & {S_{ N \to 20} } \\ \end{array} } \right] . $$

By adapting the procedure explained in PRIM, 400 coefficients are also obtained from RPRIM. Similarly, with the help of computing statistical parameters, a set of 24 elements is obtained by reducing the dimensionality of RPRIM.

### Feature scaling

Feature scaling is actually used to provide all features an opportunity to give an equal contribution to detect and predict the 4-carboxyglutamate sequencing. In this work, a standard scaler function is used within the Python environment to scale all features^30^. The standard scaler is used to scale the given data such that each feature should have mean around zero and unit variance. The standard scaling formulation is given in Eq. (11).

11 $$ {\text{Min}}{-}{\text{Max scaling}}:{ }X_{norm} = \frac{{X - X_{min} }}{{X_{max} - X_{min} }} $$

### Prediction algorithm

In this work, Multilayered Perceptron (MLP), Logistic Regression and Random Forest classifiers are applied for the prediction of 4-carboxyglutamate residue sites. MLP classifier provides better prediction which is 94% in comparison to other methods. So MLP is discussed further in detail.

The dataset has consisted of a total of 1160 sequences including 560 positive samples and 600 negative samples including 194 features. A supervised learning approach is used in this work to predict 4-carboxyglutamate residue sites. The prediction algorithm has to predict between residue sites having 4-carboxyglutamate or not.

**Figure 6:**
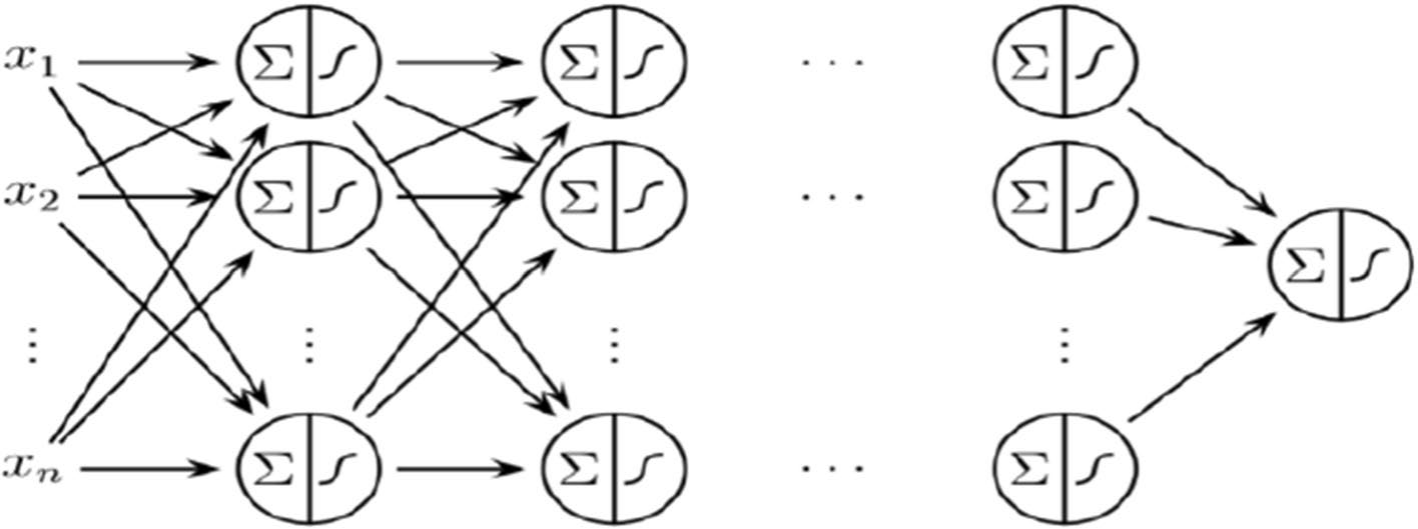
Graphical representation of MLP classifier^32^.

MLP is a feed-forward artificial neural network that is used to map input data against the most appropriate output. It is actually a directed graph consisting input and an output layer and multiple hidden layers in between them. All nodes are connected to all other nodes in the adjacent layer and therefore, it is called a fully connected network^31^. The graphical representation of the MLP classifier is given in Fig. 6.

MLP classifier consists of N neurons in the hidden layer and each neuron has R weights, which is described in the N × R matrix^33^. The input weight matrix has N elements and is denoted by I as described in Eq. (12). The functional processing of the hidden layer is explained with the help of Eqs. (12) – (14).

12 $$ {\mathbf{I}} = \left[ {\begin{array}{*{20}c} {w_{ 1,1} } & {w_{ 1,2} } & \cdots & {w_{1,R} } \\ {w_{ 2,1} } & {w_{ 2,2} } & \ldots & {w_{2,R} } \\ \vdots & \vdots & \ddots & \vdots \\ {w _{N,1} } & {w_{ N,2} } & \cdots & {w_{N,R} } \\ \end{array} } \right] $$

13 $$ {\varvec{n}}_{1} \user2{ } = {\text{I}} \cdot {\text{V}} + { }b_{1} $$

14

The sequential processing of output layer form hidden layer is explained with the help of Eqs. (15)–(17).

15 $$ {\mathbf{L}} = \left[ {\begin{array}{*{20}c} {w_{1,1} } & {w_{1,2} } & \cdots & {w_{1,S} } \\ {w_{2,1} } & {w_{2,2} } & \ldots & {w_{2,S} } \\ \vdots & \vdots & \ddots & \vdots \\ {w_{k,1} } & {w_{k,2} } & \cdots & {w_{k,S} } \\ \end{array} } \right] $$

16 $$ {\varvec{n}}_{2} \user2{ } = {\text{I}} \cdot {\varvec{a}}_{1} { } + { }b_{2} $$

17

## Results

This study is first to predict 4-carboxyglutamate residue sites. Data samples are collected and formulated as described in “Materials and methods” section. The obtained data sets had non-numeric values having a series of alphabetic values. A featured set of numeric values is obtained as explained in “Sequence logo” section. As there were a lot of variations in obtained data so feature scaling technique is used so that each feature should have equal contribution in the prediction and detection of 4-carboxyglutamate residue sites. A neural network named MLP Classifier is used to train the obtained data sets and then based on training 4-carboxyglutamate residue sites are then predicted efficiently. The process of MLP classifier is well explained using graphical representation as shown in Fig. 6 and mathematically described in Eqs. (12) – (17) respectively.

The confusion matrix obtained from the MLP classifier is described in detail in Table 1. True positive, true negative, false positive, false negative is represented as TP, TN, FP and FN respectively.

**Table 1:** Confusion matrix of the proposed model.

n = 232	Predicted	Predicted	
	No	Yes	
**Actual**			
No	T N = 106	F P = 8	114
**Actual**			
Yes	F N = 6	T P = 112	118
	112	120	

The test set consists of 232 samples where 106 negative samples out of 114 negative samples are correctly predicted and 112 positive samples out of 118 are correctly identified, as shown in Table 1.

There is a number of metrics used to validate prediction accuracy. Correct and actual prediction can be validated by Sensitivity, Specificity, Accuracy and Mathew’s Correlation Coefficient. Accuracy, Sensitivity Specificity and Mathew’s Correlation Coefficient are represented at many places in this study by Acc, Sn, Sp and Mcc respectively. Their formulation is also given below^34–36^ where Sensitivity is applied to measure the probability of the model to predict target values. Mathew’s Correlation Coefficient is used to evaluate the quality of the classification framework^37^.

18 $$ {\text{Sn}} = \frac{TP}{{TP + FN}}{ }0 \le {\text{ Sn }} \le 1{ } $$

19 $$ {\text{Sp}} = \frac{TN}{{TN + FP}}{ }0 \le {\text{ Sp }} \le 1{ } $$

20 $$ {\text{ACC}} = \frac{TP + TN}{{TP + TN + FP + FN}}{ }0 \le {\text{ Acc }} \le 1{ } $$

21 $$ {\text{Mcc}} = \frac{{\left( {TP \times TN} \right) - \left( {FP \times FN} \right)}}{{\sqrt {\left( {{\text{ T P}} + {\text{F P }}} \right)\left( {{\text{ T P}} + {\text{F N }}} \right)\left( {{\text{ T N}} + {\text{F P }}} \right)\left( {{\text{ T N}} + {\text{F N }}} \right)} }}{ } - 1 \le {\text{MCC }} \le 1{ } $$

The obtained sensitivity, specificity, accuracy and Mathew’s Correlation Coefficient are 95%, 93%, 94% and 0.88 respectively. The obtained results validate the accuracy of the prediction model. Test methods are also applied for further validation which will be elaborated in “Test methods” section.

### Test methods

There are many popular test methods in data mining and machine learning to evaluate the validity of the devised model. In this work, the independent set test, K-fold cross-validation test, and jackknife test are used to validate the devised model^38^. The independent test has 94% accuracy. K-fold cross-validation is performed with K = 10. The tenfold cross-validation test has 85% accuracy. Jackknife testing always gives you a unique value for the same dataset^8^. Jackknife testing is mostly used by an investigator to examine the quality of various predictors^38–50^. This study also uses a Jackknife test to check the quality of the predictor. The jackknife testing produced 94% accuracy. The result of all these test cases is given in Table 2. These test methods are also further explained in the coming subsections.

**Table 2:** Combined results of Multilayered Perceptron (MLP), Logistic Regression (LR) and Random Forest (RF).

	Independent set test				Self-consistency test				Tenfold cross validation test				Jack Knife test			
	Acc (%)	Sn (%)	Sp (%)	MCC	Acc (%)	Sn (%)	Sp (%)	Mc	Acc (%)	Sn (%)	Sp (%)	MCC	Acc (%)	Sn (%)	Sp (%)	MCC
MLP	94	95	93	0.88	99	99	99	0.99	85	92	79	0.71	94	93	96	0.88
LR	93	92	93	0.85	97	97	96	0.93	88	91	82	0.74	93	92	94	0.86
RF	91	90	91	0.81	89	90	88	0.78	81	86	77	0.62	88	88	89	0.76

#### Independent set test

**Figure 7:**
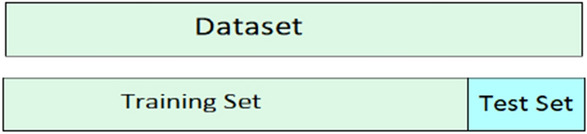
Sample dataset for independent set test.

It is the basic performance metric of the proposed model in which obtained values from a confusion matrix are used to evaluate the accuracy of the model. The dataset is split into 80% training set and 20% test set and also shown in Fig. 7.

In this study, an independent set test has 94% Acc, 95% Sn, 93% Sp and is having 0.88 Mcc achieved by Multilayered Perceptron. The results of Logistic Regression and Random Forest results can also be seen in Table 2. Acc, Sn, Sp, and Mcc is mathematically described in Eqs. (18) – (21) respectively.

**Figure 8:**
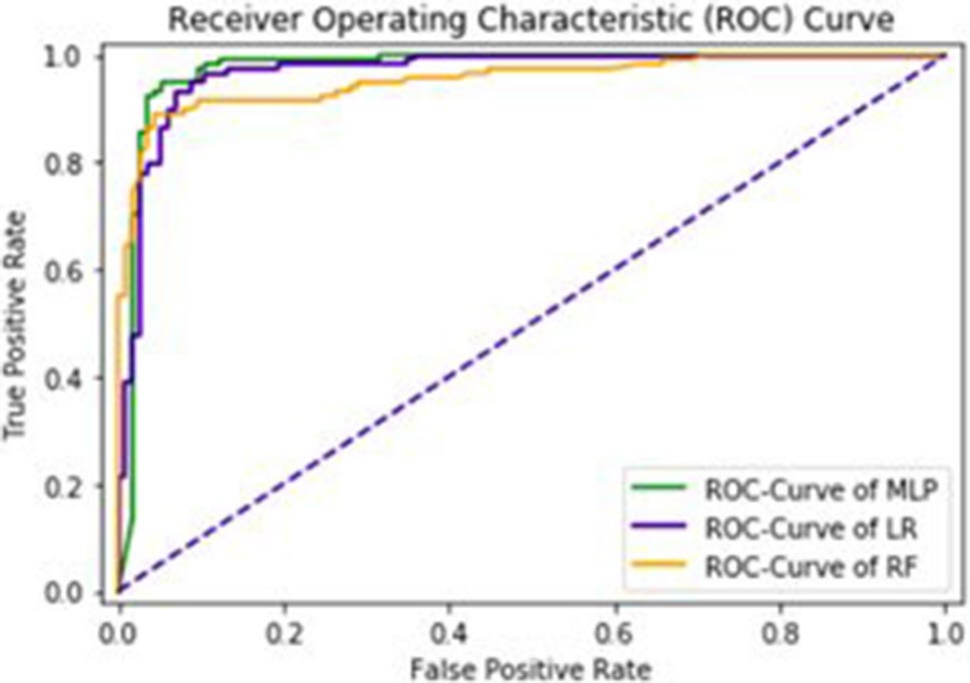
ROC-Curve of an independent set test.

The area under the curve (AUC), obtained by Multilayered Perceptron, Logistic Regression and Random Forest are 97%, 97% and 95% respectively. The F1—score obtained by Multilayered Perceptron, Logistic Regression and Random Forest are 94%, 93% and 91% respectively. It also shows correctness of classifier. ROC-Curve is given in Fig. 8.

#### Self-consistency testing

**Figure 9:**
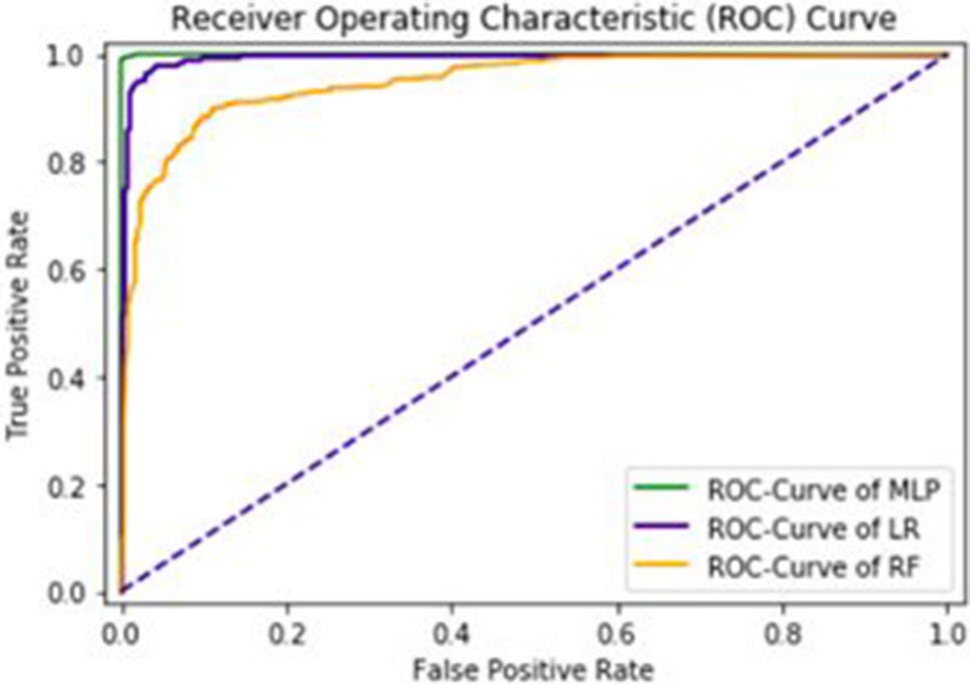
ROC-curve of self consistency test.

This technique is used to have same data for both training and testing. The results are written in Table 2 and the ROC—Curve for Multilayered Perceptron, Logistic Regression and Random Forest is shown in Fig. 9.

#### K-fold cross-validation testing

It is a sampling technique used to validate the proposed models by using a limited number of data samples. It has a single parameter k which indicates the number of groups into which the data samples should be divided^51–53^. It is mostly used to evaluate the performance of the machine learning model to invisible data^54^.

**Figure 10:**
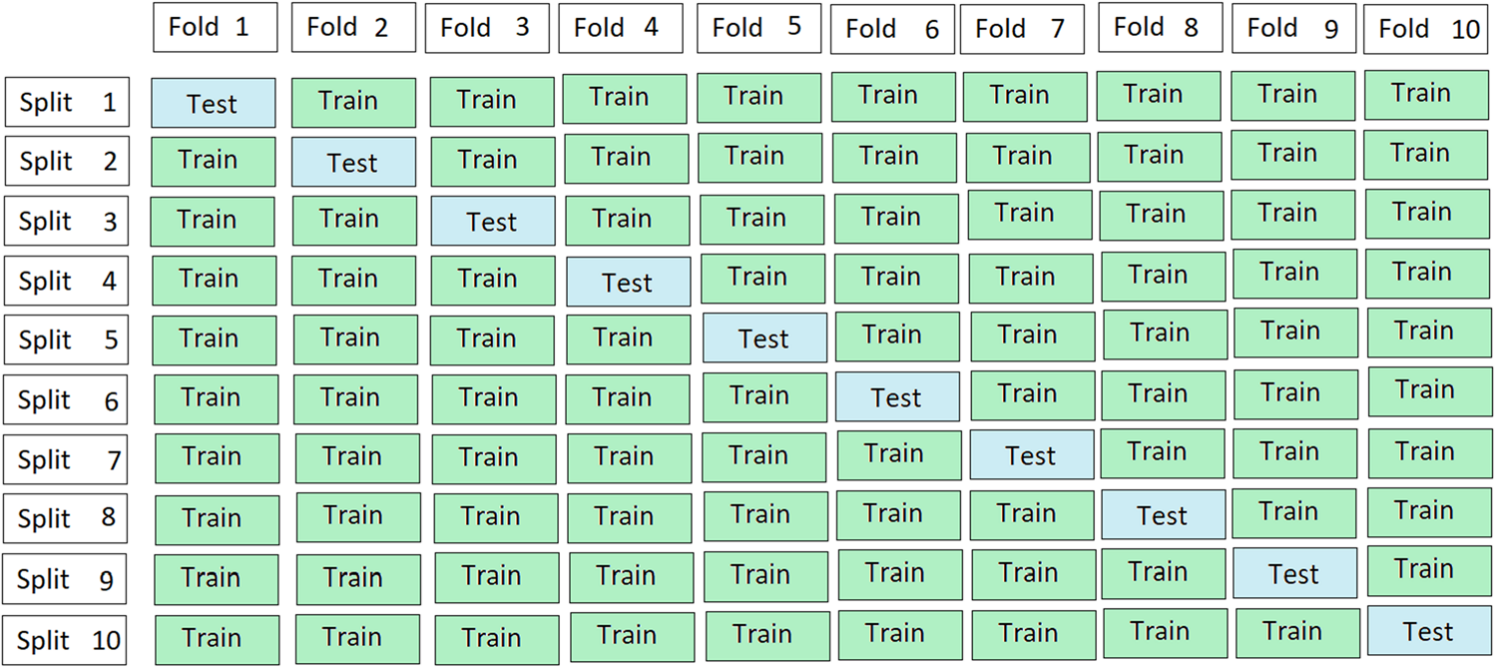
Tenfold cross validation process.

K can have any numeric value such as 5 or 10. In this work, tenfold cross validation sampling test is applied to evaluate the performance of the proposed model. The process of tenfold cross validation is also explained in Fig. 10. The data are divided into 10 equal observation sets (10 data samples). All the values such as Acc, An, Sp and Mcc are obtained for each observation set. The average of obtained accuracy for all observation sets is 85%, average sensitivity is 92%, average specificity is 79% and average Mathew’s correlation coefficient is 0.71 as given in Table 2.

**Figure 11:**
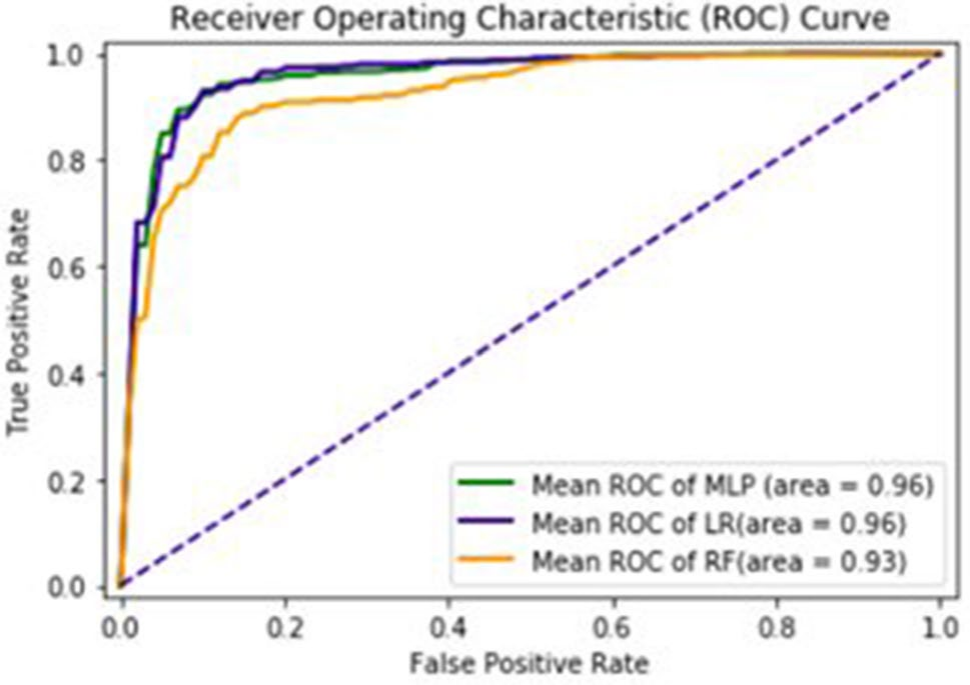
ROC-curve of tenfold cross validation test.

The detailed of ROC-Curve of MLP, LR and RF is given in Fig. 11. The AUC of MLP, LR and RF are 0.96, 0.96 and 0.93 respectively.

#### Jackknife testing

It is considered a resample technique that is mostly used to compute the bias, mean and variance^55–57^.

**Figure 12:**
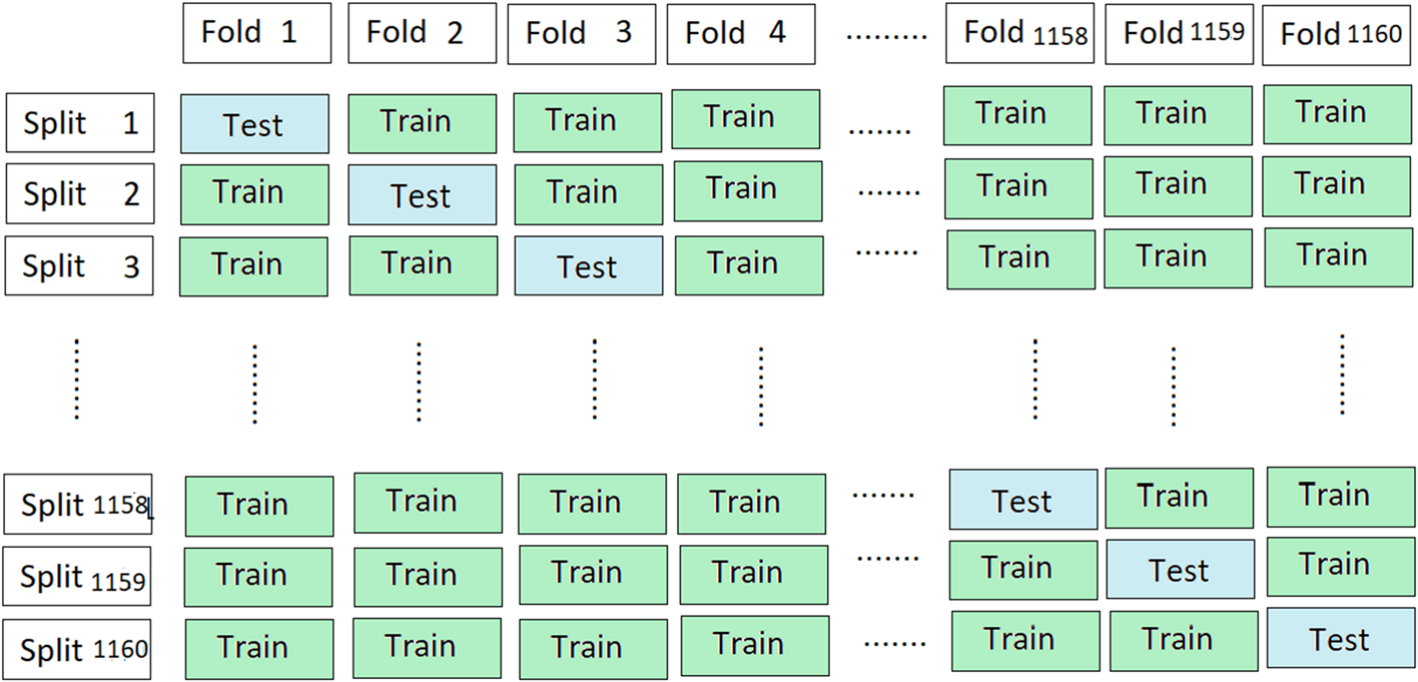
Jackknife sample test.

It evaluates the classification model sample by sample. The proposed classification model is validated on each sample using Jackknife testing and an average is computed of all the obtained results based on each sample. The process is also explained in Fig. 12. Overall observation samples are 1160 and therefore classification model is run 1160 times with obtained accuracy 94% along with sensitivity 93%, specificity 96% and Mathew’s Correlation Coefficient 0.88.

The sequences are taken from a universal resource of protein (www.UniProt.org) through an advanced search. The chosen sequencings are streams of alphabets. It is difficult to process these sequences directly through the machine learning algorithm as they are unable to provide quantification measures. In order to address this issue, the feature vector is extracted from chosen sequences in a way that it has a strong correlation among features. In order to scale the obtained features, a standard normalization technique is used. A multilayered perceptron classifier is then applied to learn hidden patterns within observed features. Based on the said intelligent learning, observed features are going to be trained first which will then be a groundbreaking step for prediction. The validation of the proposed algorithm is carried out using a confusion matrix which is given in Table 1. Acc, Sn, Sp, and Mcc are estimated using FP, FN, TP, and TN within the confusion matrix which are 94%, 95%, 93% and 0.88 respectively as given in Table 2 and area under the curve is 0.97. Three different Machine learning algorithms are applied such as Multilayer Perceptron (MLP), Logistic Regression (LR) and Random Forest (RF). Four different types of tests are applied such as an independent set test, self-consistency test, cross validation test, and jackknife test. In this study it is clear from ROC curves that MLP is a better approach. The obtained results using different test cases validates the authenticity of our proposed model that it performs well even if the data set has large variations. Along with independent set test, self-consistency test, tenfold cross-validation test and jackknife test also obtained very good results as given in Table 2.

## Conclusion

Glutamate is an important type of common alpha-amino acid. 4-Carboxyglutamic acid is produced by a post-translational carboxylation of glutamic acid residues. This study is conducted to predict 4-carboxyglutamate following Chou’s 5 steps rule. An MLP, RF and LR classification frameworks are adopted for the prediction of 4-carboxyglutamate residue sites. The accuracy of the independent set test, self-consistency test, tenfold cross-validation test, and Jackknife testing were determined to be 94%, 99%, 85% and 94%, respectively. A properly devised model will help in accurate detection of 4-carboxyglutamate which may be useful in evaluation of blood clotting, bone proteins, bone resorption, osteoporosis, papilloma and plaque atherosclerotic statuses.

## References

[1] Danbolt NC (2001). Glutamate uptake. Prog. Neurobiol..

[2] Lee CA (2014). Textbook of Hemophilia.

[3] Horava SD, Peppas NA (2017). Recent advances in hemophilia B therapy. Drug Deliv. Transl. Res..

[4] Suttie JW (1985). Vitamin K-dependent carboxylase. Annu. Rev. Biochem..

[5] Burnier JP, Borowski M, Furie BC, Furie B (1981). Gamma-carboxyglutamic acid. Mol. Cell. Biochem..

[6] Pacifici R (1987). Spontaneous release of interleukin 1 from human blood monocytes reflects bone formation in idiopathic osteoporosis. Proc. Natl. Acad. Sci..

[7] Malm J, Cohen E, Dackowski W, Dahlback B, Wydro R (1990). Expression of completely gamma-carboxylated and beta-hydroxylated recombinant human vitamin-K-dependent protein S with full biological activity. Eur. J. Biochem..

[8] Gijsbers BL, Haarlem LJV, Soute BA, Ebberink RH, Vermeer C (1990). Characterization of a Gla-containing protein from calcified human atherosclerotic plaques. Arteriosclerosis.

[9] Glutamic Acid. in*National Center for Biotechnology Information. PubChem Compound Database*. https://pubchem.ncbi.nlm.nih.gov/compound/Glutamic-acid. Accessed 26 Apr 2020.

[10] -Carboxyglutamic acid. in*National Center for Biotechnology Information. PubChem Compound Database*. https://pubchem.ncbi.nlm.nih.gov/compound/4-Carboxyglutamic-acid#section=Structures. Accessed 26 Apr 2020.

[11] Chou KC (2011). Some remarks on protein attribute prediction and pseudo amino acid composition. J. Theor. Biol..

[12] Chou KC (2001). Using subsite coupling to predict signal peptides. Protein Eng..

[13] Arif M, Hayat M, Jan Z (2018). iMem-2LSAAC: A two-level model for discrimination of membrane proteins and their types by extending the notion of SAAC into Chou's pseudo amino acid composition. J. Theor. Biol..

[14] Contreras-Torres E (2018). Predicting structural classes of proteins by incorporating their global and local physicochemical and conformational properties into general Chous PseAAC. J. Theor. Biol..

[15] Feng P-M, Chen W, Lin H, Chou K-C (2013). iHSP-PseRAAAC: Identifying the heat shock protein families using pseudo reduced amino acid alphabet composition. Anal. Biochem..

[16] Javed F, Hayat M (2018). Predicting subcellular localization of multi-label proteins by incorporating the sequence features into Chous PseAAC. Genomics.

[17] Krishnan SM (2018). Using Chous general PseAAC to analyze the evolutionary relationship of receptor associated proteins (RAP) with various folding patterns of protein domains. J. Theor. Biol..

[18] Sankari ES, Manimegalai D (2018). Predicting membrane protein types by incorporating a novel feature set into Chous general PseAAC. J. Theor. Biol..

[19] Khan YD, Rasool N, Hussain W, Khan SA, Chou KC (2018). iphosY-PseAAC: Identify phosphotyrosine sites by incorporating sequence statistical moments into PseAAC. Mol. Biol. Rep..

[20] Chou KC (2015). Impacts of bioinformatics to medical chemistry. Med. Chem..

[21] Chou KC (2001). Impacts of bioinformatics to medical using pseudo-amino acid composition. Proteins.

[22] Khan YD, Ahmad F, Anwar MW (2012). A neuro-cognitive approach for iris recognition using backpropagation. World Appl. Sci. J..

[23] Khan YD, Ahmed F, Khan SA (2013). Situation recognition using image moments and recurrent neural networks. Neural Comput. Appl..

[24] Butt H, Khan SA, Jamil H, Rasool N, Khan YD (2016). A prediction model for membrane proteins using moments based features. Biomed. Res. Int..

[25] Butt H, Rasool N, Khan YD (2016). A treatise to computational approaches towards prediction of membrane protein and its subtypes. J. Membr. Biol..

[26] Khan YD (2014). An efficient algorithm for recognition of human actions. Sci. World J..

[27] Khan YD, Khan SA, Ahmad F, Islam S (2014). Iris recognition using image moments and k-means algorithm. Sci. World J..

[28] Khan YD, Rasool N, Hussain W, Khan SA, Chou KC (2018). iPhosT-PseAAC: Identify phosphothreonine sites by incorporating sequence statistical moments into PseAAC. Anal. Biochem..

[29] Akmal MA, Rasool N, Khan YD (2017). Prediction of N-linked glycosylation sites using position relative features and statistical moments. PLoS ONE.

[30] sklearn.preprocessing.StandardScaler. scikit. https://scikit-learn.org/stable/modules/generated/sklearn.preprocessing.StandardScaler.html. Accessed 8 Mar 2020.

[31] Wan S, Liang Y, Zhang Y, Guizani M (2018). Deep multi-layer perceptron classifier for behavior analysis to estimate Parkinson’s disease severity using smartphones. IEEE Access..

[32] Gajoui KE, Allah FA, Oumsis M (2015). Diacritical language OCR based on neural network: Case of Amazigh language. Procedia Comput. Sci..

[33] Zhai X, Ali AAS, Amira A, Bensaali F (2016). MLP neural network based gas classification system on Zynq SoC. IEEE Access..

[34] Chen J, Liu H, Yang J, Chou K-C (2007). Prediction of linear B-cell epitopes using amino acid pair antigenicity scale. Amino Acids.

[35] Xu Y, Ding J, Wu L-Y, Chou K-C (2013). iSNO-PseAAC: Predict cysteine S-nitrosylation sites in proteins by incorporating position specific amino acid propensity into pseudo amino acid composition. PLoS ONE.

[36] Chen W, Feng P-M, Lin H, Chou K-C (2013). iRSpot-PseDNC: Identify recombination spots with pseudo dinucleotide composition. Nucleic Acids Res..

[37] Porter J, Berkhahn J, Zhang L, Tran QN, Arabnia H (2015). A comparative analysis of read mapping and indel calling pipelines for next-generation sequencing data. Emerging Trends in Computational Biology, Bioinformatics, and Systems Biology.

[38] Chou K-C, Zhang C-T (1995). Prediction of protein structural classes. Crit. Rev. Biochem. Mol. Biol..

[39] Ali F, Hayat M (2015). Classification of membrane protein types using voting feature interval in combination with Chou’s pseudo amino acid composition. J. Theor. Biol..

[40] Zhou G-P, Doctor K (2002). Subcellular location prediction of apoptosis proteins. ProteinsStruct. Funct. Bioinform..

[41] Mondal S, Pai PP (2014). Chou’s pseudo amino acid composition improves sequence-based antifreeze protein prediction. J. Theor. Biol..

[42] Feng K-Y, Cai Y-D, Chou K-C (2005). Boosting classifier for predicting protein domain structural class. Biochem. Biophys. Res. Commun..

[43] Nanni L, Brahnam S, Lumini A (2014). Prediction of protein structure classes by incorporating different protein descriptors into general Chou’s pseudo amino acid composition. J. Theor. Biol..

[44] Shen H-B, Yang J, Chou K-C (2007). Euk-PLoc: An ensemble classifier for large-scale eukaryotic protein subcellular location prediction. Amino Acids.

[45] Wu Z-C, Xiao X, Chou K-C (2011). iLoc-Plant: A multi-label classifier for predicting the subcellular localization of plant proteins with both single and multiple sites. Mol. BioSyst..

[46] Dehzangi A (2015). Gram-positive and gram-negative protein subcellular localization by incorporating evolutionary-based descriptors into Chou’s general PseAAC. J. Theor. Biol..

[47] Qiu W-R, Xiao X, Chou K-C (2014). iRSpot-TNCPseAAC: Identify recombination spots with trinucleotide composition and pseudo amino acid components. Int. J. Mol. Sci..

[48] Kumar R, Srivastava A, Kumari B, Kumar M (2015). Prediction of β-lactamase and its class by Chou’s pseudo-amino acid composition and support vector machine. J. Theor. Biol..

[49] Chen J, Long R, Wang X-L, Liu B, Chou K-C (2016). dRHP-PseRA: Detecting remote homology proteins using profile-based pseudo protein sequence and rank aggregation. Sci. Rep..

[50] Ahmad K, Waris M, Hayat M (2016). Prediction of protein submitochondrial locations by incorporating dipeptide composition into Chou’s general pseudo amino acid composition. J. Membr. Biol..

[51] Duchesnay, E. & Löfstedt, T. *Statistics and Machine Learning in Python Release 0.2*. (2018).

[52] Adams, R. P. *Model Selection and Cross Validation Evaluation Hygiene: The Train/Test Split,* 1–8.

[53] Anguita, D. Ghelardoni, L. Ghio, A. Oneto, L & Ridella, S. The ‘K’ in K-fold cross validation. in*European Symposium on Artificial Neural Networks, Computational Intelligence and Machine Learning*, 441–446 (2012).

[54] Rodríguez JD, Pérez A, Lozano JA (2010). Sensitivity analysis of k-fold cross validation in prediction error estimation. IEEE Trans. Pattern Anal. Mach. Intell..

[55] Chapter 8 Bootstrap and Jackknife Estimation of Sampling. https://www.stat.washington.edu/jaw/COURSES/580s/581/LECTNOTES/ch8.pdf. Accessed 24 May 2019.

[56] G Protein-Coupled Receptor 172A (GPR172A) ELISA Kit. Human GPR172A ELISA Kit (ABIN5654457). https://www.antibodies-online.com/kit/5654457/GProtein-CoupledReceptor172AGPR172AELISAKit/. Accessed 8 Mar 2020.

[57] Lavergne C (1995). A Jackknife method for estimation of variance components. Statistics.

